# PBE-GGA predicts the B8↔B2 phase boundary of FeO at Earth’s core conditions

**DOI:** 10.1073/pnas.2304726120

**Published:** 2023-07-03

**Authors:** Zhen Zhang, Yang Sun, Renata M. Wentzcovitch

**Affiliations:** ^a^Department of Applied Physics and Applied Mathematics, Columbia University, New York, NY 10027; ^b^Department of Physics, Iowa State University, Ames, IA 50011; ^c^Department of Earth and Environmental Sciences, Columbia University, New York, NY 10027; ^d^Lamont-Doherty Earth Observatory, Columbia University, Palisades, NY 10964; ^e^Data Science Institute, Columbia University, New York, NY 10027

**Keywords:** thermodynamics, anharmonicity, strongly correlated material, electronic structure, Earth’s core

## Abstract

FeO is a crucial component of the Earth’s core, and its thermodynamic properties are essential to developing more accurate core models. It is also a notorious correlated insulator in the NaCl-type (B1) phase at ambient conditions. It undergoes two polymorphic transitions at 300 K before it becomes metallic in the NiAs-type (B8) structure at ~100 GPa. Although its phase diagram is not fully mapped, it is well established that the B8 phase transforms to the CsCl-type (B2) phase at core pressures and temperatures. Here, we report a successful ab initio calculation of the B8↔B2 phase boundary in FeO at Earth’s core pressures. We show that fully anharmonic free energies computed with the Perdew–Burke–Ernzerhof-generalized gradient approximation coupled with thermal electronic excitations reproduce the experimental phase boundary within uncertainties at *P* > 255 GPa, including the largely negative Clapeyron slope of –52 MPa/K. This study validates the applicability of a standard density functional theory functional to FeO under Earth’s core conditions and demonstrates the theoretical framework that enables complex predictive studies of this region.

## Significance

FeO is a compound of great interest in materials physics and geophysics. It has a complex pressure–temperature (*P,T*) phase diagram, including subtle structural and magnetic transitions. Currently, it is challenging for prevailing theoretical approaches for strongly correlated materials, such as FeO at low *P,T*s, to address anharmonic vibrations. We use density functional theory–based molecular dynamics and lattice dynamics calculations to demonstrate the dynamic and thermodynamic stability of an experimentally observed but theoretically unidentified B2-type phase of FeO at high *P,T*s. We show that this phase is unstable at low *T*s, but is stabilized at high *P,T*s by anharmonic effects and thermal electronic excitations. This study establishes a theoretical framework for studying FeO and related Fe alloys at the Earth’s core conditions.

FeO is one of the major constituents of Earth and other terrestrial planets. It is not only the iron end-member of ferropericlase [(Mg_1-_*_x_*Fe*_x_*)O], the second most abundant phase in the Earth’s lower mantle, but also a significant alloying component in the Earth’s core (1). As a classic “correlated” oxide, phase relations in FeO are also of great interest in condensed matter physics. Like ferropericlase, FeO undergoes a spin state change under pressure (2, 3). FeO exhibits rich phenomenology at high pressures and temperatures (*P,T*), e.g., polymorphic, magnetic, and insulating–metallic transitions. Such phase changes control fundamental material properties of the Earth, e.g., its thermal and electrical conductivity and magnetic susceptibility, to mention a few.

FeO has an enigmatic phase diagram (e.g., see ref. 4). It is stable in the cubic NaCl–type (B1) structure at ambient conditions. Under compression at room temperature, B1 undergoes a phase transition to a rhombohedral B1 (rB1) structure above 16 GPa (5). While rB1 is believed to be the ground state (6) at low temperatures and low pressures, B1 remains stable at higher temperatures along the geotherm throughout the mantle (2, 7–9). A further change to the NiAs-type (B8) structure was observed above 90 GPa at 600 K (10). The B1↔rB1 and rB1↔ B8 phase boundaries have been measured at and above 300 K (10, 11). A B1↔ B8 phase boundary has also been measured at high temperatures up to ~240 GPa, a typical outer core pressure (2, 7–9). In addition, the monoclinic B1 (12, 13) and the inverse B8 phases (11, 14) have also been observed at low temperatures.

Besides these five crystal structures, FeO’s CsCl-type (B2) structure (15, 16) has been identified by experimental measurements at higher *P,T*. Unlike MgO, CaO, SrO, and BaO, which transform directly from the B1 to the B2 structure, FeO has the intermediate and partially covalent/metallic B8 phase (4, 10) up to ~3,800 K and ~240 GPa (15). The direct B1↔ B2 transformation in FeO occurs only above this temperature (15), with a B1, B2, and B8 triple point expected near these conditions. So far, only one experimental study has reported measurements of B2-related phase boundaries (15). Electronic structure and spin states of the B1 (2, 3, 8, 17–20) and B8 (20–24) phases have often been investigated, given the theoretical challenge of dealing with the interplay between electronic correlation and structural phase transitions.

Here, we perform ab initio calculations of the B8↔ B2 boundary at ~150 to 400 GPa, a relevant pressure range for the Earth’s core, and *T* > 1,000 K. Under such extreme conditions, anharmonicity is fundamental in determining dynamic and thermodynamic stabilities, especially for the B2 phase [e.g., the analogous *bcc* phase of elemental metals (25–29) and the B2 phase of binary compounds (30)]. The high-spin to low-spin (LS) and insulator-to-metal transitions in FeO happen at ~120 GPa (24, 31), a typical mantle pressure. A recent density functional plus dynamical mean-field theory (DFT+DMFT) study (20) confirmed the LS and metallic (delocalized) electronic state in both B2 and B8 phases under core conditions. These observations suggest that DFT-based molecular dynamics (DFT-MD) could address the B8↔ B2 phase competition.

## Results and Discussion

**Fig. 1.**
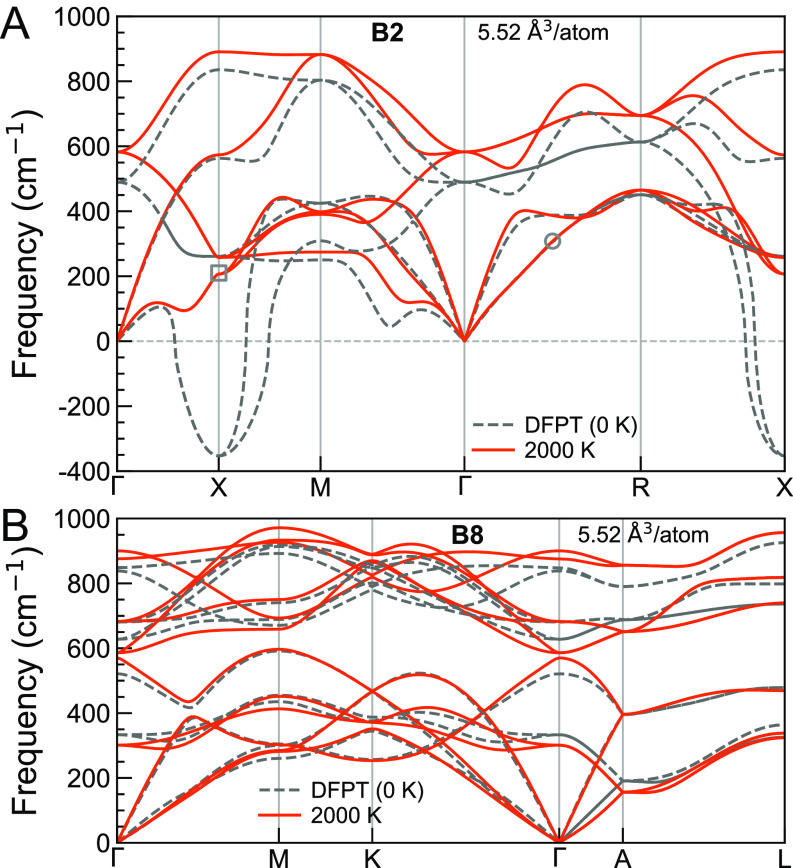
Harmonic (dashed gray curves) and anharmonic phonon dispersions at 2,000 K (solid orange curves) for (*A*) B2 and (*B*) B8 phases at *V* = 5.52 Å^3^/atom.

We first address harmonic phonon dispersions at ionic temperature *T* = 0 K in both B2 and B8 phases using density functional perturbation theory (DFPT) (32). DFT and DFPT calculations were performed using the PAW method (33) as implemented in Quantum ESPRESSO (34). We used the Perdew–Burke–Ernzerhof (PBE) (35)-generalized gradient approximation (GGA) to compute the exchange–correlation energy. Phonons were calculated on 4×4×4 and 4×4×2 **q**-meshes in the B2 and B8 structures, respectively. Fig. 1 *A* and *B* shows harmonic phonon dispersions (dashed gray curves) for these phases. The B2 phase displays unstable modes around phonon wave vector $q\;=\;X\left(0,\frac{1}{2},0\right)$ , while the B8 phase’s phonons are all stable. The harmonic phonon instability in the B2 phase is not out of expectation. A similar soft mode behavior in the *bcc* phase of many elemental metals (25–29) drives the *bcc* to *hcp* phase transition at low temperatures. The B2 and B8 phases of FeO are analogous to the elemental *bcc* and *hcp* phases, respectively (*SI Appendix*, Fig. S1). Note that though B8 FeO shares the same $P6_{3}/mmc$ space group with the elemental *hcp* phase, the atomic layers stacking in B8 ($A^{Fe}B^{O}A^{Fe}C^{O}\;A^{Fe}B^{O}A^{Fe}C^{O}$) differs from that in elemental *hcp* (AB AB). Imaginary frequencies at 0 K indicate that phonon–phonon interactions are critical in the dynamic stabilization of this structure, and entropic effects at high temperatures can stabilize it thermodynamically. Conventional harmonic phonons or quasiharmonic free energies cannot address the lattice dynamics and thermodynamic properties of this B2 phase.

We employ the phonon quasiparticle (PHQ) approach (36, 37) to address the high-temperature anharmonicity of FeO under core *P,T* conditions. This approach assumes that a system with fully interacting phonons can be simplified as an effective system with noninteracting PHQs (38, 39). Each PHQ can be described by two parameters, renormalized frequency, ${\tilde{\omega }}_{qs}$ , and linewidth, $\Gamma_{qs}$ . A PHQ is numerically defined by mode-projected velocity autocorrelation function (VAF) (36, 37),

[1] $$\langle V_{qs}\left(0\right)V_{qs}\left(t\right)\rangle =\lim_{\tau \to \infty }\frac{1}{\tau }\int_{0}^{\tau }V_{qs}^{*}\left(t^{'}\right)V_{qs}\left(t^{'}+t\right)dt^{'},$$

where Vqst=∑i=1NMiviteiq·Ri·e^qs i is the mass-weighted and **q***s*-mode-projected velocity. $M_{i}$ , $R_{i}$ , and $v_{i}$ $\left(i=1,\ldots ,N\right)$ are atomic mass, crystallographic atomic coordinate, and atomic velocity computed by DFT-MD of an *N*-atom supercell, respectively. e^qs i is the harmonic phonon eigenvector obtained at electronic temperature $T_{el}=T$ in the DFT-MD (29, 38, 39), where **q** should be commensurate with the supercell size and *s* labels the phonon branches at each **q**. For a well-defined PHQ, its VAF assumes an exponentially decaying cosine form $A_{qs}cos\left({\tilde{\omega }}_{qs}t\right)e^{-\Gamma_{qs}t}$ , where $A_{qs}$ is the initial oscillation amplitude. The well-defined VAF’s power spectrum ${\left|\int_{0}^{\infty }\langle V_{qs}(0)V_{qs}(t)\rangle e^{i\omega t}dt\right|}^{2}$ assumes a Lorentzian line shape with a single peak at ${\tilde{\omega }}_{qs}$ and a linewidth $\Gamma_{qs}$ (36, 37).

To compute PHQs, ab initio molecular dynamics (AIMD) (40) simulations were performed. Simulations details are found in the *Materials and Methods* section. Well-defined PHQs obtained on the AIMD-sampled **q**-mesh enable calculations of renormalized, i.e., anharmonic phonon dispersion using Fourier interpolation (36, 37). Fig. 1 *A* and *B* shows the anharmonic phonon dispersions obtained at *T* = 2,000 K (solid orange curves) for the B2 and B8 phases, respectively. The unstable mode with imaginary harmonic frequency at $q\;=\;X\left(0,\frac{1}{2},0\right)$ in the B2 phase stiffens drastically at high temperatures (see the gray square in Fig. 1*A* and *SI Appendix*, Fig. S2). The anharmonic phonon dispersions are free of imaginary frequencies, indicating that B2 FeO is stabilized dynamically by phonon–phonon interactions at high temperatures (29, 37, 41). Frequency renormalization in the B8 phase is not as significant as in the B2, yet also not negligible. Thus, the following free energy calculations use temperature-dependent anharmonic phonon spectra (see an example in *SI Appendix*, Fig. S3) for both phases. The anharmonic phonon spectra were evaluated on much denser **q**-meshes (20×20×20 for B2 and 20×20×10 for B8) via Fourier interpolation (36, 37) to approximate the thermodynamic limit (N→∞).

**Fig. 2.**
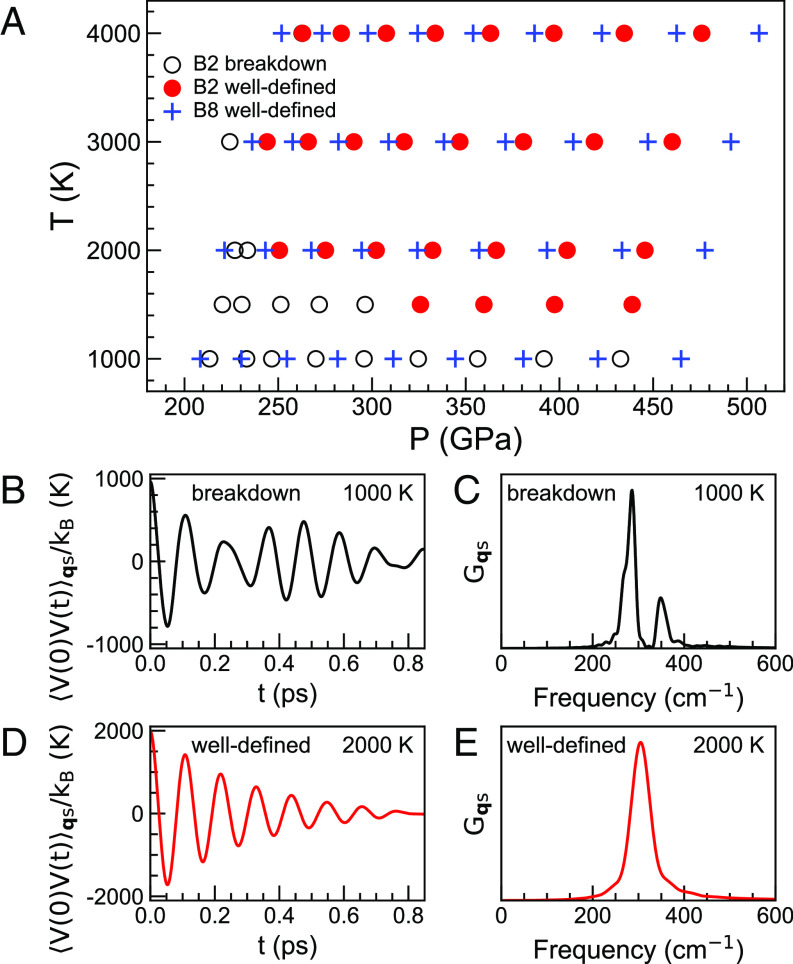
(*A*) *P,T* conditions covered by AIMD simulations. Hollow circles indicate conditions where the B2 structure is unstable and/or B2 PHQs are not well defined. Red circles indicate a dynamically stable B2 phase with well-defined PHQs. Blue crosses indicate a dynamically stable B8 phase with well-defined PHQs. Pressures are ensemble averages from AIMD simulations. (*B*) Mode-projected VAF of the transverse acoustic mode at $q\;=\;\left(\frac{1}{4},\frac{1}{4},\frac{1}{4}\right)$ (gray circle in Fig. 1*A*) with a harmonic frequency of 304 cm^−1^ for B2 at *V* = 5.52 Å^3^/atom and *T* = 1,000 K. (*C*) shows the corresponding power spectrum. (*D*) Mode-projected VAF of the same mode at *T* = 2,000 K, and (*E*) the corresponding power spectrum.

We performed AIMD simulations for both B8 and B2 phases at the *P,T* conditions indicated in Fig. 2*A*. The corresponding *V,T* conditions are shown in *SI Appendix*, Fig. S4. The B8 phase is dynamically stable at all studied *P,T*s, while the B2 phase shows structural and/or PHQ instabilities at certain low *P,T*s. For instance, at *T* = 1,000 K and *P* = ~213 GPa (*V* = 5.88 Å^3^/atom), the eight-fold coordinated Fe in the starting B2 structure transforms to a sixfold coordinated structure after thermal equilibration (*SI Appendix*, Fig. S5). By removing the B2 lattice constraints, a complete phase transition to B8 is realized. Compressed from ~213 to ~270 GPa (*V* = 5.52 Å^3^/atom) at *T* = 1,000 K, the structure no longer shows the B2→ B8 transition. PHQs in the B2 phase are still not well defined at this *P,T*. Fig. 2*B* shows the 1,000 K VAF for the transverse acoustic mode at $q\;=\;\left(\frac{1}{4},\frac{1}{4},\frac{1}{4}\right)$ (gray circle in Fig. 1*A*), which exhibits a pattern far distinct from an exponentially decaying cosine form. The corresponding power spectrum shows two peaks (Fig. 2*C*), indicating the breakdown of a well-defined B2 phase PHQ (29, 37). This behavior signals the tendency of atoms to displace from the B2 equilibrium sites and the B2 structure to distort. In contrast, this mode is stable at 2,000 K (~275 GPa at the same *V* = 5.52 Å^3^/atom), as indicated by the well-defined PHQ with an exponentially decaying VAF (Fig. 2*D*) and a single Lorentzian-shaped peak in the power spectrum (Fig. 2*E*). As shown in Fig. 2*A*, higher *P,T*s systematically stabilize the B2 phase.

The extensive AIMD results indicated in Fig. 2*A* enable Gibbs free energy calculations on an equal footing for the B2 and B8 phases in a large *P,T* range. With Fourier interpolated anharmonic phonon spectra, the vibrational entropy can be obtained in the thermodynamic limit within the phonon gas model (36, 37, 42),

[2] $$S_{vib}\left(T\right)=k_{B}\sum_{qs}\left[\left(n_{qs}+1\right)\ln\left(n_{qs}+1\right)-n_{qs}\ln n_{qs}\right],$$

where $n_{qs}={\left[\exp(\hbar {\tilde{\omega }}_{qs}\left(T\right)/k_{B}T)\;-\;1\right]}^{-1}$. ${\tilde{\omega }}_{qs}\left(T\right)$ at *T* not directly sampled by AIMD was obtained by fitting calculated ${\tilde{\omega }}_{qs}$’s at several AIMD-sampled temperatures and constant volume to a second-order polynomial in *T* (29, 36, 41). The Helmholtz free energy at constant volume can be obtained by integrating both the electronic and vibrational entropies (29, 41),

[3] $$\begin{array}{c} F(T)=E(T_{0})-T_{0}\left[S_{\text{el}}(T_{0})+S_{\text{vib}}(T_{0})\right]\\ \;\;\;\;-\overset{T}{\underset{T_{0}}{\int }}\left[S_{\text{el}}(T^{\prime })+S_{\text{vib}}(T^{\prime })\right]dT^{\prime }, \end{array}$$

where $T_{0}$ is a reference temperature at which the structure is dynamically stable and all PHQs are well defined and $E\left(T_{0}\right)$ and $S_{el}\left(T_{0}\right)$ are time-averaged internal energy and electronic entropy obtained from AIMD at $T_{0}$, respectively. The choice of $T_{0}$ does not change the resulting thermodynamics, so we set $T_{0}=4,000\;K$. $S_{el}\left(T\right)$ at constant volume was computed as the ensemble average at AIMD-sampled temperatures and fit to a second-order polynomial in *T* (29). Contrary to $S_{vib}$ that suffers from finite-size effects and requires Fourier interpolation, $S_{el}$ and E are more insensitive to the simulation cell size (39), and AIMD ensemble averages converge well.

**Fig. 3.**
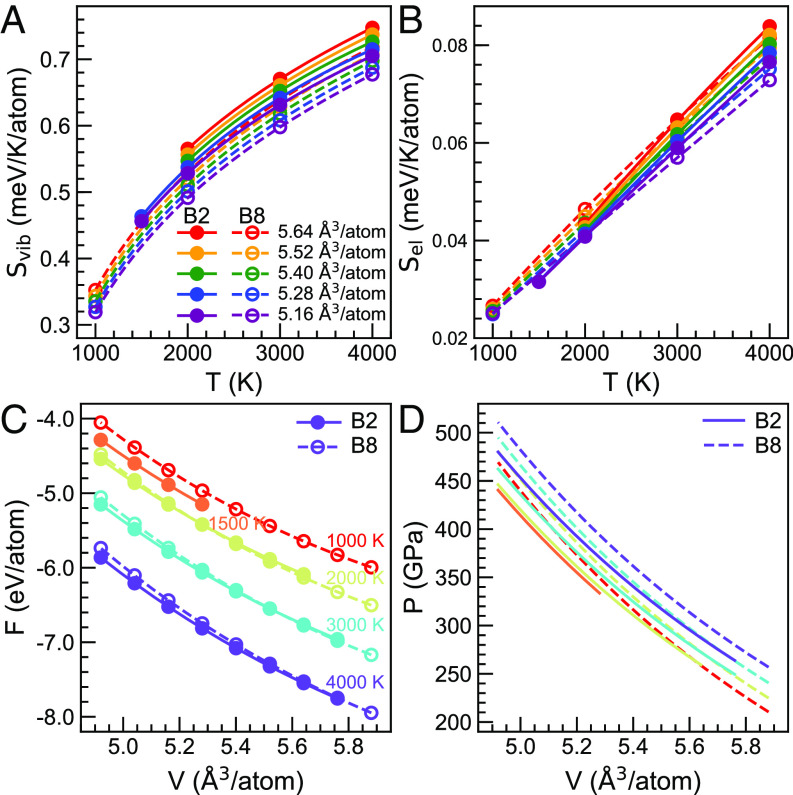
(*A*) Vibrational entropy $S_{vib}\left(V,T\right)$ and (*B*) electronic entropy $S_{el}\left(V,T\right)$ versus *T* at different *V*s. (*C*) Helmholtz free energy F(V,T) and (*D*) pressure P(V,T) versus *V* at different *T*s. B2 is shown by solid circles and solid curves, and B8 is shown by open circles and dashed curves. Circles indicate the V,T s sampled by the AIMD simulations.

At the same *V,T* conditions, $S_{vib}$ is always larger for B2 than for B8 (Fig. 3*A*). The generally lower renormalized frequencies in B2 (*SI Appendix*, Fig. S3) contribute to its larger entropy and thermodynamic stability with respect to B8 at higher temperatures. $S_{el}$ varies nearly linearly with temperature (Fig. 3*B*), and a quadratic fitting for $S_{el}\left(T\right)$ is sufficiently accurate (29). $S_{el}$’s of both phases are similar but much smaller than $S_{vib}$ (Fig. 3 *A* and *B*). Therefore, $S_{vib}$ dominates entropic effects on the free energy. Calculated $F\left(V,T\right)$ s are shown in Fig. 3*C*. Isothermal equations of state (EOS) were computed by fitting $F\left(V\right)$ to a third-order finite strain expansion. Pressures calculated as P=-∂F∂VT are shown in Fig. 3*D*. At the same *P,T* conditions, the stable B2 phase volume is always smaller than the B8 volume, and the difference $\Delta \left(PV\right)={\left(PV\right)}_{B8}-{\left(PV\right)}_{B2}$ increases with pressure (*SI Appendix*, Fig. S6), which contributes to the enthalpic stabilization of B2 at high pressures. We thereby predict a negative Clapeyron slope (dPdT=ΔSΔV) for the phase boundary.

**Fig. 4.**
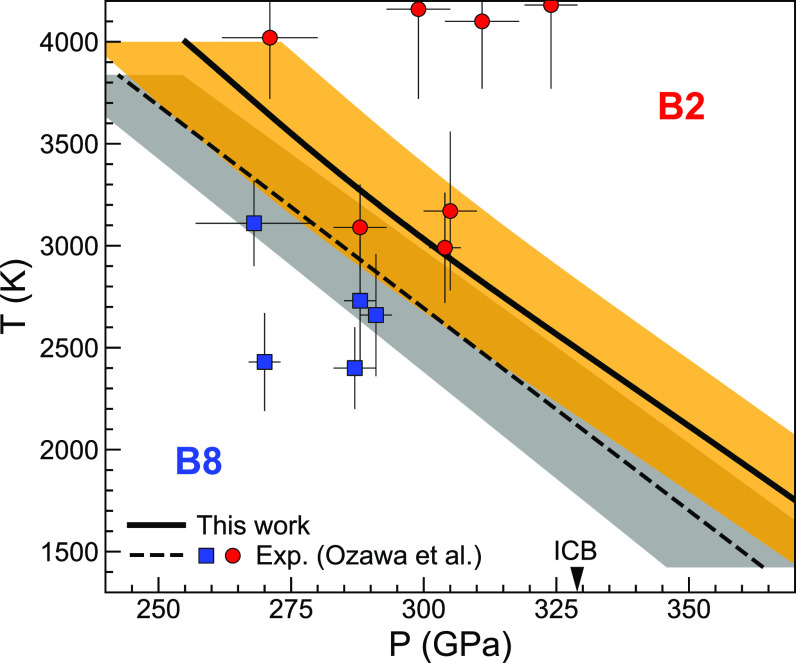
B8↔ B2 phase boundary of FeO. The solid black curve is the calculated phase boundary, and the shaded orange area indicates the computational uncertainty. The X-ray diffraction measurements (15) for B2 (red circles with error bars) and B8 (blue squares with error bars) phases are shown for comparison. The dashed line is the experimental phase boundary (15), and the shaded gray area indicates a recent estimation of its uncertainty by Kei Hirose resulting from the choice of the Fe EOS (45) as a pressure scale. The arrow indicates the inner-core boundary (ICB) pressure.

The Gibbs free energy was calculated as $G\left(P,T\right)=$ $F\left(V,T\right)+P\left(V,T\right)V$ utilizing the fitted EOS. Comparing the Gibbs free energies of both phases (*SI Appendix*, Fig. S7), we obtain the B8↔ B2 phase boundary shown in Fig. 4. The uncertainty in our free energy and phase boundary calculations is estimated as follows: *SI Appendix*, Fig. S8 shows the dependence of the Helmholtz free energy difference, ΔF, between the two phases on the **k**-mesh sampling. We consider the results on a much more computationally expensive 3×3×3 **k**-mesh in the 128-atom supercell to be fully converged since it differs by less than 1 meV/atom from the 2×2×2 **k**-mesh result. Hence, the difference between the Γ-point and 3×3×3 **k**-mesh results is ~9 meV/atom. This is the adopted uncertainty in ΔF arising from **k**-mesh sampling. The uncertainty in ΔF arising from fluctuations in AIMD simulations is estimated by conducting five parallel runs at a constant volume and 4,000 K, which gives ~0.1 meV/atom. Note that the dense **q**-mesh sampled in the entropy calculation (Eq. **2**) mimics a sufficiently large supercell (16,000 atoms) calculation in the thermodynamic limit. These combined effects give an uncertainty in ΔF of ~9 meV/atom, and this value is passed to ΔG. This estimated uncertainty in the free energy difference compares well with the ~10 meV/atom uncertainty reported in the calculation of the melting curve of iron (38) using the same PHQ approach. It is also similar to uncertainties in other free energy difference calculations using thermodynamic integration (43). The uncertainty in the PV term given by fitting the isothermal EOS at several volumes is a second-order effect, thus was disregarded here. The free energy uncertainty leads to an average uncertainty of ±18 GPa in the transition pressure, shown as the shaded orange area in Fig. 4. The accuracy of our prediction, however, is better than the precision. The difference between the predicted and measured (15) phase boundaries is ~5% of the pressure, which is a typical uncertainty by ab initio calculations (e.g., see ref. 44). The error bars in Fig. 4 label the reported experimental uncertainties in *P,T* (15). A recent estimation of the uncertainty in the experimental transition pressure (15) resulting from the choice of the Fe EOS (45) as a pressure scale is also ~5% of the pressure by one of the authors of the experimental study (15), Kei Hirose, and is shown as the shaded gray area in Fig. 4. As such, the level of agreement between our predictions and measurements of this phase boundary is excellent.

In the temperature range shown (*T* < 4,000 K), the B8↔ B2 transition occurs at *P* > 255 GPa. The melting properties of FeO are beyond the scope of this study. At the inner-core boundary (ICB) pressure, 329 GPa (15), the calculated transition temperature is 2,496 K. Under such conditions, the B8→ B2 transition is accompanied by a 1.5% density increase. Like the analogous *hcp*↔*bcc* transition in elemental metals (28, 39, 46), the Clapeyron slope of the B8↔ B2 transition is also negative. The B8↔ B2 phase boundary is nearly linear with an average Clapeyron slope of –52 MPa/K, which is in excellent agreement with that measured by experiments, –50 MPa/K (15).

In summary, we have investigated the B8↔ B2 phase boundary of FeO at high *P,T* conditions of the Earth’s core with ab initio calculations. We computed anharmonic free energies in the thermodynamic limit using PHQ dispersions. The calculated phase boundary agrees with experimental observations (15) within uncertainties. The successful calculation of the B8↔ B2 phase boundary demonstrates that the PBE-GGA functional describes well energy differences between the B8 and B2 phases of FeO at these high *P,T* conditions. We might attribute this success to two factors: 1) FeO is metallic and nonmagnetic at the relevant high *P,T*s. A comparison between the electronic density of states calculated with PBE-GGA and DFT+DMFT (20) (*SI Appendix*, Fig. S9) shows qualitatively similar electronic structures near the Fermi level; 2) at the relevant high *P,T*s, anharmonic effects on the B2 phase are dominant, and the vibrational entropy differences between the two phases are also well described by PBE-GGA. The success of PBE-GGA in studying this metal-to-metal transition is further supported by a recent work by Kaplan and Perdew (47), which argues that the short range of the exact exchange–correlation hole in a metal, as a consequence of perfect long-range screening, can make GGAs and Laplacian-level meta-GGAs more accurate than full meta-GGAs or hybrid functionals for metals.

The double challenge of treating the strongly correlated FeO at low *P,T*s and the strongly anharmonic nature of the B2 phase at high *P,T*s has inhibited direct calculations of this phase boundary previously. Some prevailing theoretical approaches for FeO include DFT+U and DFT+DMFT. However, DFT+U incorrectly delivers an insulating state for the B8 phase at core conditions. Also, DFT+U is a static approximation (U is a function of position), which hinders its use in AIMD for (anharmonic) vibrational properties (48). Also, DFT+DMFT is not practical for AIMD (48). Therefore, the present results establish a theoretical framework based on AIMD with standard DFT functional and PHQ approach for materials’ study under extreme conditions. This theoretical framework solves a critical problem for FeO at high *P,T* conditions that could not be addressed by other approaches such as DFT+U or DFT+DMFT.

Such a theoretical framework also enables future predictive studies of the Fe–FeO system at these high *P,T* conditions, which is key to understanding the debated problem of oxygen partitioning between the liquid and the solid regions of the Earth’s core and their density deficits. Owing to the low oxygen solubility in iron at low pressures, it has been proposed that the liquid outer core must crystallize into Fe without oxygen to form the dense inner core (49). Only the liquid outer core is believed to contain oxygen (50), and such oxygen concentration difference might account for the density jump across the ICB. However, the recent discovery of Fe-rich Fe–O (Fe*_n_*O) compounds experimentally and theoretically at core pressures (51) has changed this view. It suggests that the solid inner core could also incorporate a significant amount of O and, thus, a more complex origin of the outer and inner core density deficit contrast. The experiments in ref. 51 were conducted at high *P,T* conditions where B8-FeO is stable. The present study justifies the use of PBE-GGA for ab initio computations of B8-FeO and close-packed Fe*_n_*O phases (51) at relevant conditions. Considering the electronic density of states, chemical bonding, and charge transfer in the Fe*_n_*O phases are related to those in their end-members, i.e., FeO and Fe (51), the present study validates the use of PBE-GGA in future ab initio studies of the Fe*_n_*O compounds with a B2-FeO end-member at inner core conditions including full anharmonicity. The present study also opens the door for future calculations of thermodynamic properties and crystallization of the O-bearing outer core liquid phase with PBE-GGA. Liquid properties obtained from such ab initio calculations will help the development of more accurate core convection and geodynamo models.

## Materials and Methods

### AIMD Simulations.

AIMD simulations were performed using the PAW PBE as implemented in Vienna ab initio simulation package (40). The electronic temperature ($T_{el}$) was set the same as the ionic temperature (*T*) using the Mermin functional (52, 53). FeO was simulated with 128-atom supercells (4×4×4 for B2 and 4×4×2 for B8) with a Γ **k**-point sampling and a kinetic energy cutoff of 400 eV. The supercells were sufficiently large to converge the harmonic part of interatomic force constants. Thus, they should also be sufficiently large to converge the anharmonic part since the anharmonic part of interatomic force constants has shorter ranges than those of the harmonic one (37, 54). Simulations were conducted in the *NVT* ensemble on a series of volumes between 4.92 and 5.88 Å^3^/atom and temperatures between 1,000 and 4,000 K controlled by Nosé thermostat (55, 56). Each simulation ran for 50 ps, sufficiently long to converge PHQ parameters, with a time step of 1 fs.

## Supplementary Material

Appendix 01 (PDF)

## Data Availability

All study data are included in the article and/or *SI Appendix*.
