# Supplementary material for: PBE-GGA predicts the B8↔B2 phase boundary of FeO at Earth’s core conditions
Source: Proc Natl Acad Sci U S A. 2023 Jul 3;120(28):e2304726120. doi: 10.1073/pnas.2304726120 (PMC10334785; doi:10.1073/pnas.2304726120)
Supplement: Supplementary file 1 — Appendix 01 (PDF) [file pnas.2304726120.sapp.pdf]

PNAS

Supporting Information for

PBE-GGA predicts the B8↔B2 phase boundary of FeO at Earth's core conditions

Zhen Zhang[a,1], Yang Sun[a,b,1], Renata M. Wentzcovitch[a,c,d,*]
[a]Department of Applied Physics and Applied Mathematics, Columbia University, New York, NY 10027, USA.
[b]Department of Physics, Iowa State University, Ames, IA 50011, USA.
[c]Department of Earth and Environmental Sciences, Columbia University, New York, NY 10027, USA.
[d]Lamont-Doherty Earth Observatory, Columbia University, Palisades, NY 10964, USA.
[1]Z.Z. and Y.S. contributed equally to this work.
*corresponding author: Renata M. Wentzcovitch
Email: rmw2150@columbia.edu

This PDF file includes:

Figures S1 to S9
SI References

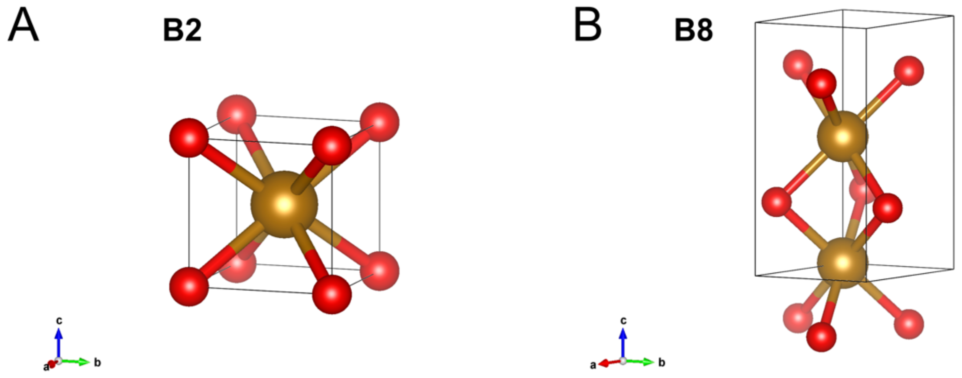

Fig. S1. The crystal structure of (*A*) CsCl-type B2 phase and (*B*) NiAs-type B8 phase of FeO. Iron is shown in golden, and oxygen in red. B2 has a 2-atom primitive cell in the cubic lattice, and B8 has a 4-atom primitive cell in the hexagonal lattice.

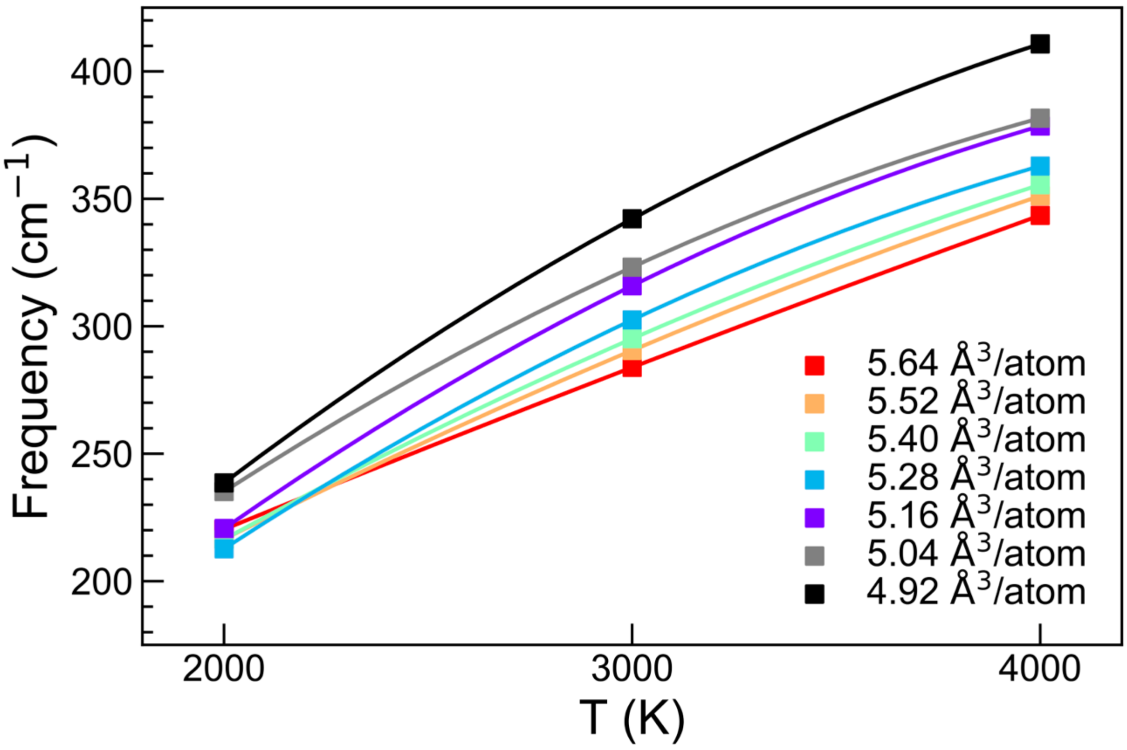

Fig. S2. Temperature-dependent renormalized frequency of the transverse phonon mode at $\mathbf{q} = \mathbf{X}(0, \frac{1}{2}, 0)$ (gray square in Fig. 1(a)) with imaginary harmonic frequency for B2 calculated at constant volume. All soft modes at different volumes acquire real renormalized frequencies and stiffen drastically with increasing temperature.

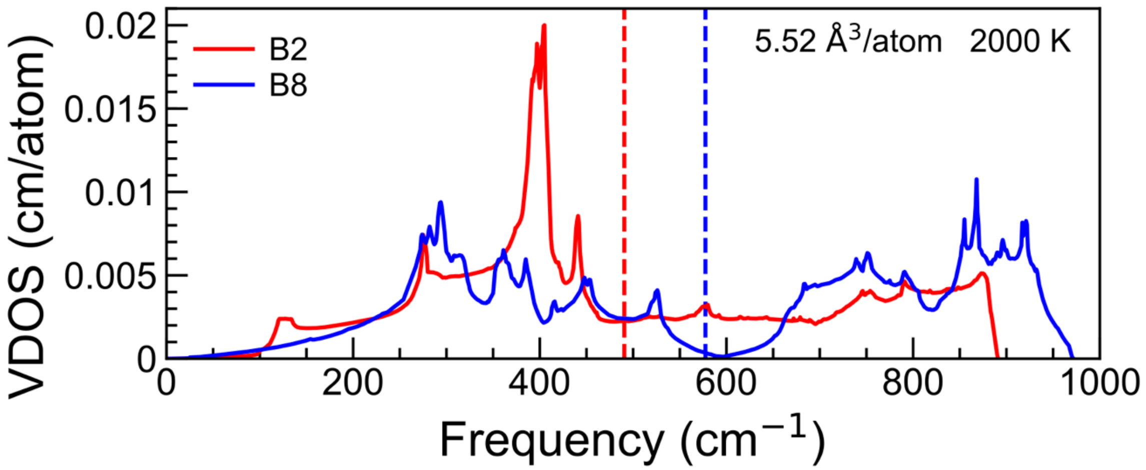

Fig. S3. Anharmonic vibrational density of states (VDOS) for B2 (red curve) and B8 (blue curve) at 2000 K and V = 5.52 Å^3/atom. The vertical dashed lines indicate the average renormalized frequencies.

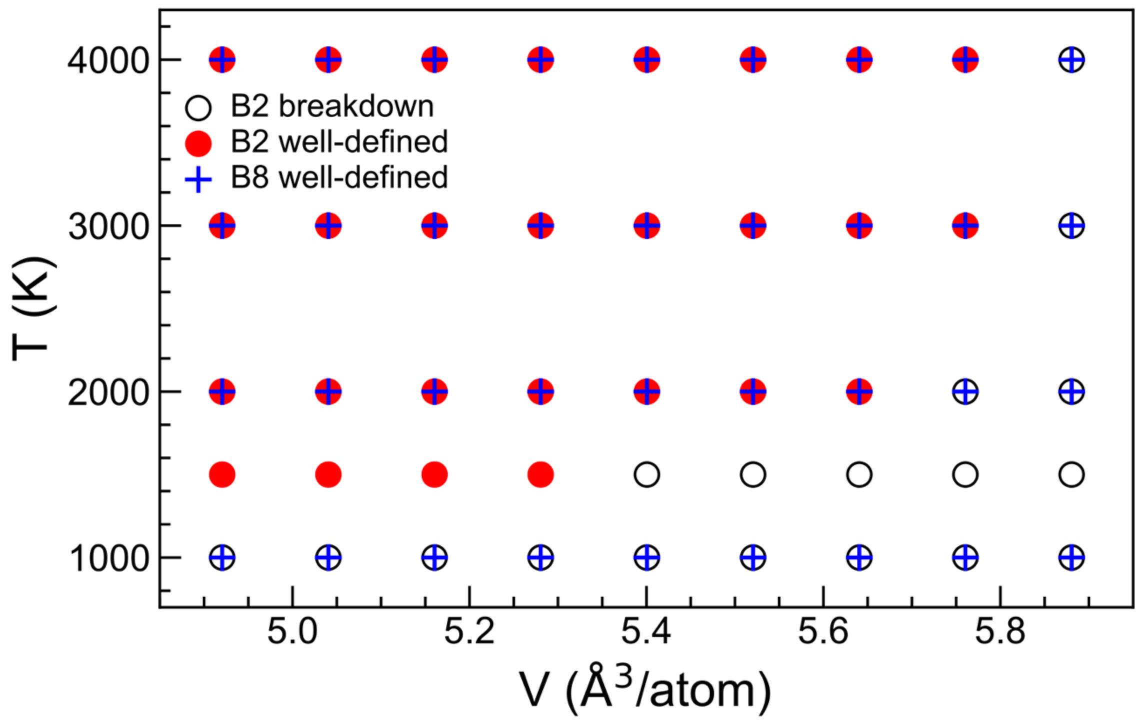

Fig. S4. *V,T* conditions covered in the AIMD simulations. Conditions at which structures and/or phonon quasiparticles for B2 break down are indicated by hollow circles. Conditions at which both structures and phonon quasiparticles are well-defined are indicated by red circles and blue crosses for B2 and B8, respectively.

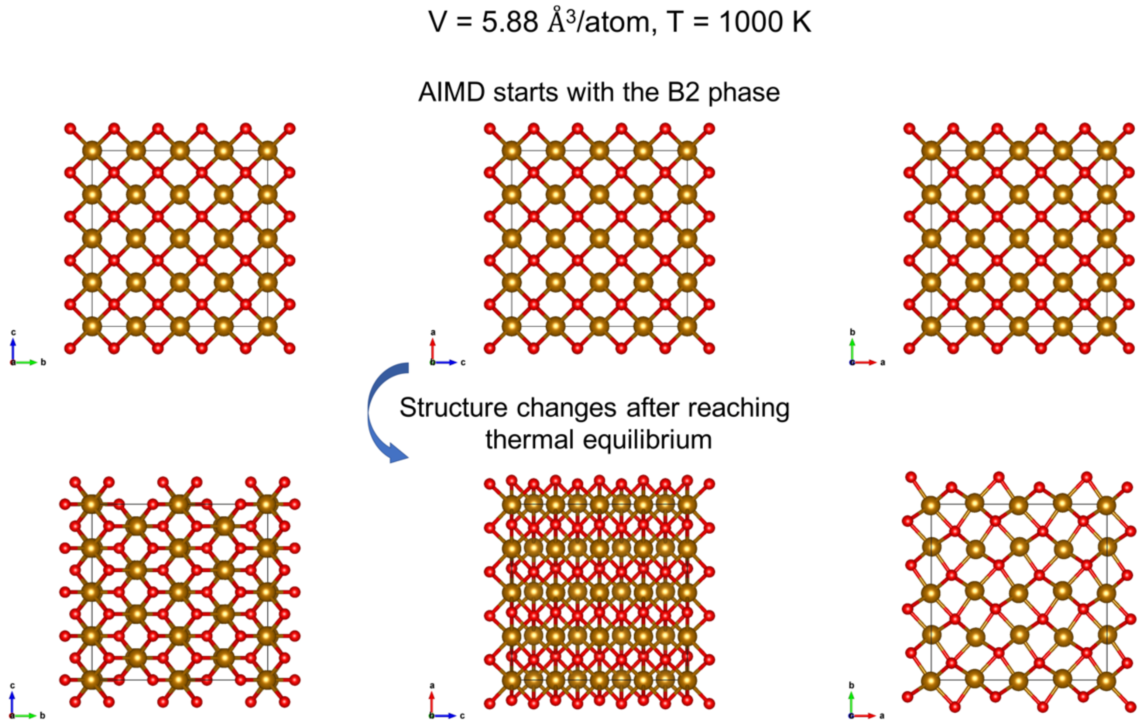

Fig. S5. Time-averaged supercell structure in the B2 lattice after reaching thermal equilibrium in the AIMD simulated at $V = 5.88$ Å^3/atom and $T = 1000$ K. The simulation starts with the B2 phase, but the B2 structure is dynamically unstable at this condition. Iron is shown in golden, and oxygen in red. Left, middle, and right are the three views of the same supercell, respectively.

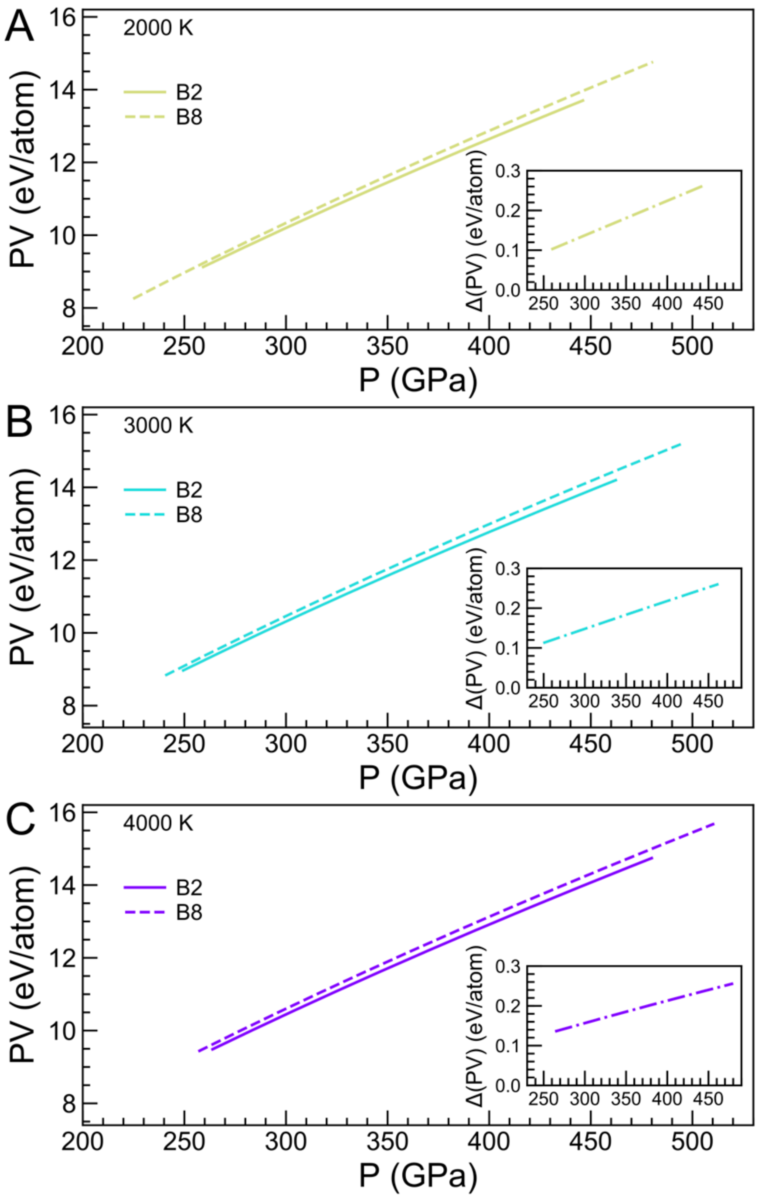

Fig. S6. PV versus pressure for B2 (solid curves) and B8 (dashed curves) at (A) 2000, (B) 3000, and (C) 4000 K, respectively. Inserts: $\Delta(PV) = (PV)_{B8} - (PV)_{B2}$ as a function of pressure at each temperature.

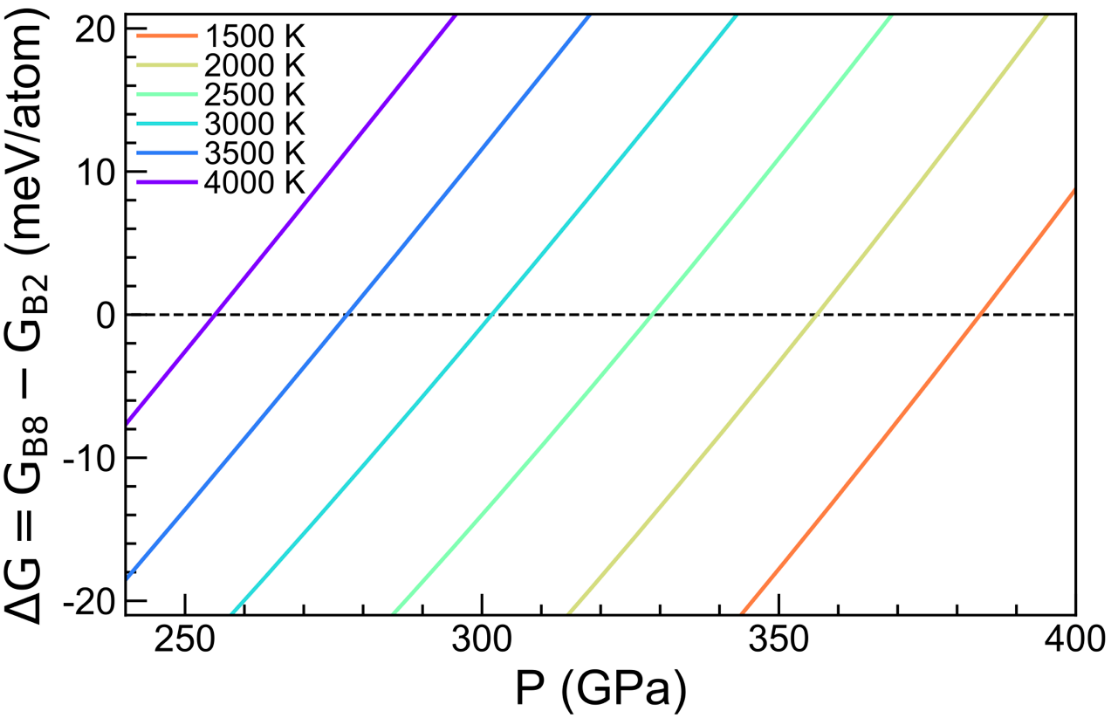

Fig. S7. Gibbs free energy difference $\Delta G = G_{\mathrm{B8}} - G_{\mathrm{B2}}$ as a function of pressure at different temperatures.

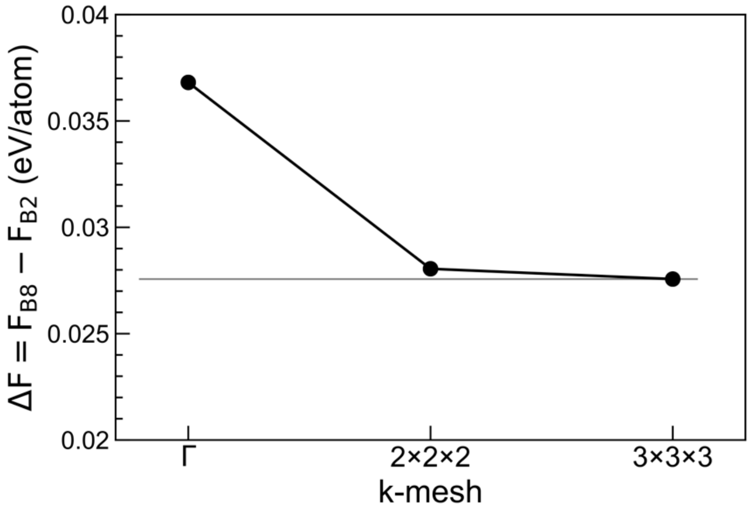

Fig. S8. Helmholtz free energy difference $\Delta F = F_{\mathrm{B8}} - F_{\mathrm{B2}}$ calculated with Γ, 2×2×2, and 3×3×3 **k**-mesh sampling for the 128-atom supercell at V = 5.52 Å^3/atom and T = 4000 K. The computational error in **k**-mesh sampling is ~9 meV/atom by comparing ΔF between Γ and 3×3×3 **k**-mesh sampling. The ΔF variance between 2×2×2 and 3×3×3 **k**-mesh sampling is <1 meV/atom.

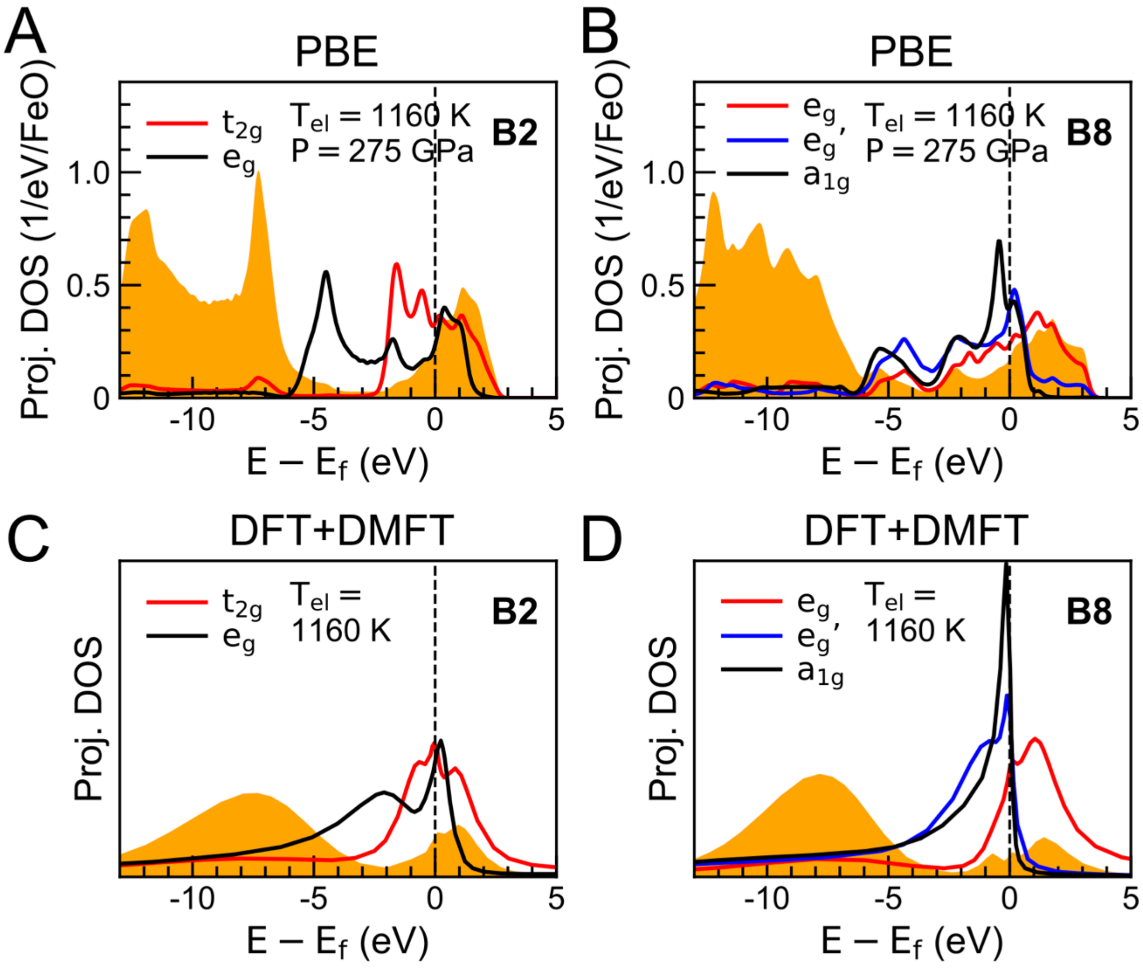

Fig. S9. Projected electronic density of states (DOS) for (*A*) B2 and (*B*) B8 FeO calculated by the PBE-GGA at T_{el} = 1160 K and static P = 275 GPa. Contributions from Fe 3*d* states are shown by solid curves, and those from O 2*p* states are shown by shaded orange areas. Projected electronic DOS for (*C*) B2 and (*D*) B8 FeO calculated by the DFT+DMFT (1) at T_{el} = 1160 K and relevant pressures are exhibited for comparison.

SI References

1. E. Greenberg *et al.*, Phase transitions and spin-state of iron in FeO at the conditions of Earth's deep interior. *arXiv:2004.00652* (2020).
